# Effect of single follow-up home visit on readmission in a group of frail elderly patients – a Danish randomized clinical trial

**DOI:** 10.1186/s12913-019-4528-9

**Published:** 2019-10-25

**Authors:** Maurice A. Lembeck, Lau C. Thygesen, Birgitte Dreyer Sørensen, Lisbeth Lumby Rasmussen, Ellen A. Holm

**Affiliations:** 1 Department of Internal Medicine, Nykøbing Falster Hospital, Fjordvej 15, 4800 Nykøbing Falster, Denmark; 2 National Institute of Public Health, University of Southern Denmark, Copenhagen K, Denmark; 3 Department of Quality and Development, Sorø, Region Zealand Denmark; 4 Nykøbing Falster Hospital, Nykøbing Falster, Denmark

**Keywords:** Discharge planning, Readmission, Frailty, Elderly, Clinical trial

## Abstract

**Background:**

Unplanned hospital admissions are costly and prevention of these has been a focus for research for decades. With this study we aimed to determine whether discharge planning including a single follow-up home visit reduces readmission rate. The intervention is not representing a new method but contributes to the evidence concerning intensity of the intervention in this patient group.

**Methods:**

This study was a centrally randomized single-center controlled trial comparing intervention to usual care with investigator-blinded outcome assessment. Patients above the age of 65 were discharged from a single Danish hospital during 2013–2014 serving a rural and low socioeconomic area. For intervention patients study and department nurses reviewed discharge planning the day before discharge. On the day of discharge, study nurses accompanied the patient to their home, where they met with the municipal nurse. Together with the patient they reviewed cognitive skills, medicine, nutrition, mobility, functional status, and future appointments in the health care sector and intervened if appropriate.

Readmission at any hospital in Denmark within 8, 30, and 180 days after discharge is reported. Secondary outcomes were time to first readmission, number of readmissions, length of stay, and readmission with Ambulatory Care Sensitive Conditions, visits to general practitioners, municipal services, and mortality.

**Results:**

One thousand forty-nine patients aged > 65 years discharged from medical, geriatric, emergency, surgical or orthopedic departments met inclusion criteria characteristic of frailty, e.g. low functional status, need of more personal help and multiple medications. Among 945 eligible patients, 544 were randomized. Seven patients died before discharge. 56% in the intervention group and 54% in the control group were readmitted (*p* = 0.71) and 23% from the intervention group and 22% from the control group died within 180 days. There were no significant differences between intervention and control groups concerning other secondary outcomes.

**Conclusions:**

There was no effect of a single follow-up home visit on readmission in a group of frail elderly patients discharged from hospital.

**Trial registration:**

https://clinicaltrials.gov (identifier NCT02318680), retrospectively registered December 11, 2014.

## Background

With a growing number of elderly individuals, most western societies are struggling with growing budgets for health care and are seeking ways to make use of economic and human resources as rational and effective as possible. Unplanned admissions are costly and prevention of these has been a focus for research for decades.

A large number of admissions are deemed “avoidable” for different reasons: 1) if the condition for which the patient is admitted could have been managed in primary care, and 2) if the patient is readmitted within 30 days from discharge implying that initial hospital treatment and discharge planning was insufficient [1].

Specific diagnoses have been defined as ambulatory care sensitive conditions (ACSC) and therefore judged as potentially avoidable [2, 3]. The concept of ACSC diagnoses was originally described in 1976 and since then it has been used as a performance indicator in the primary health care sector in several countries among these Denmark [3, 4]. No universal definition of ACSC diagnoses exists, but common diagnoses such as dehydration, constipation, pneumonia, urinary tract infection, gastroenteritis, fragility fractures, iron deficiency anemia, pressure ulcers, and hospitalization due to social causes have been suggested [5, 6]. The idea behind the concept is that if prevention and care in the primary health care sector is sufficient, hospital admissions may be prevented. It is important to bear in mind that the concept of ACSC is theoretical and to our knowledge, no clinical trials have demonstrated hospitalizations due to ACSC-diagnoses to have a greater potential for being prevented than hospitalizations due to other diagnoses. Compared to other European countries Denmark has a high frequency of hospitalizations due to ACSC conditions [7].

Readmission within 30 days after discharge from hospital is used as a quality indicator for hospital treatment and for primary care. In the USA, the 30 days readmission rate among Medicare patients lies stable about 20%, and since 2012, hospitals with a higher readmission rate than expected are financially penalized [8–11]. Several risk factors for readmission have been identified. A systematic literature review found functional status, illness severity, comorbidity, polypharmacy, diagnosis or presenting illness and age to be associated with risk of readmission [12]. Another recent study identified adverse events during the index hospitalization, previous admission and a diagnosis of vascular or liver diseases as risk factors for readmission [13]. Admission due to ACSC conditions as well as readmissions are highly related to sociodemographic characteristics including income, educational level, ethnicity, belonging to a minority, living alone, and age [2, 5, 14–24]. Several theoretical readmission prediction models have been developed but their clinical usefulness have not been demonstrated [25, 26].

Previous studies aimed to prevent hospital readmissions have diverging results, probably due to different study design and patient populations [27]. With the present study we aim to complement the evidence concerning the effect of discharge planning by focusing on a single follow-up home visit administered to frail elderly patients living in a rural area of Denmark. With a growing elderly population we found it sensible to test a single follow-up home visit in order to examine if a less intensive intervention could possibly influence readmission rate. In this study, we considered patients frail when they had a number of disabilities or known risks for readmission, i.e. we have used a frailty concept based on accumulated disabilities [28]. Thus, our study is not testing a new method but it aims to investigate if a less intensive intervention may be efficient.

## Methods

### Design and setting

The study is a single-centre, 1:1 parallel-group individual patient randomized controlled trial stratified by municipality and discharging hospital department followed for 180 days. The study was conducted and reported in accordance with the CONSORT guidelines [29]. The study took place at the Hospital of Nykøbing Falster, which serves a rural district with a population of approximately 150,000 persons in three municipalities: Guldborgsund, Lolland, and Vordingborg. The area is characterized by a low socioeconomic level and many elderly inhabitants. The Danish health care system is financed through income tax where every citizen has universal, free and equal access to the health system. The municipalities have a district nurse system, which offers home care to mainly elderly people when needed.

### Participants and recruitment

Inclusion criteria were age 65 or older (during the first 13 months of the study the age limit used was age 78 or older, but due to few participants we extended the age spectrum), discharge with any diagnosis from the Medical, Geriatric, Emergency, Surgical or Orthopedic departments at Nykøbing Falster Hospital from 1 January 2013 to 31 December 2014. The original choice of 78 as age criteria was based on statistical analysis of readmissions in our region showing a readmission frequency of 21% in this age group. Minimum 3 out of 9 medical and social conditions had to be met: cognitive and psychiatric disorders, drug or alcohol abuse, lack of social network (recent loss of spouse, ill spouse, living alone), low level of functioning, multiple medications (6 or more drugs), hospital contacts within 6 months before index hospitalization, falls history, suspicion of housing conditions that hamper the patient in his daily activities. The last criteria was not specified but was considered fulfilled if for instance home care employees or family told about chaotic conditions in a patients home like for instance rooms stuffed with furniture or old newspapers. The inclusion criteria are not classical frailty criteria, but were developed in a local group of practitioners including geriatric specialists and nurses from hospital as well as from the municipalities. Exclusion criteria were discharge between 4 pm and 8 am on weekdays; discharge during weekends; planned readmission; need of terminal care; neither patient nor family (in the case of patients with cognitive problems) were capable to give informed consent; and former participation in the study. All included patients provided informed consent.

### Randomization

Patients fulfilling the inclusion criteria were eligible and were referred to two project nurses at the hospital (the follow-home team). A project nurse obtained informed consent from the patient, or if a patient was not able to give informed consent, from the family and general practitioner. As soon as the discharge date was known, patients were randomized to intervention or control group. Discharge was usually planned the day before. Randomization was conducted via a computer-generated randomization sequence using Trial Partner. Department staff was blinded to randomization until the day before discharge.

### Intervention

The project nurse and the nurse from the discharging department reviewed the patient’s hospitalization and discharge plans on the day before discharge. On the day of discharge, the project nurse accompanied the patient to the patient’s home where they met the municipal nurse. Together with the patient and in the patient’s own surroundings the two nurses performed a structured assessment reviewing cognitive skills, medicine, nutrition, home environment, mobility, level of functioning and future appointments in the health care sector. The assessment was followed by an intervention based on the findings in the assessment. If for instance the nurses found cognitive dysfunction, the patient would be referred to skilled nursing specialists on dementia or if there were questions concerning use of drugs the patients GP would be consulted. Another intervention could be minor adjustments in the home environment, i.e. removing carpets or furniture. The project nurses were nurses with several years of experience in elderly care.

Patients in the control group were given usual care. This implied communication between hospital and municipality and general practitioner by means of electronic communication concerning hospitalization, advice on medications, home care, and rehabilitation in the municipality.

### Outcomes

The predefined primary outcome was unplanned readmission at any hospital in Denmark within 180 days after the discharge of the index admission, however readmissions during the first 8 and 30 days period were analysed as well.

The predefined secondary outcomes were time to first readmission, number of readmissions, length of stay, and readmission with an ACSC diagnosis, as defined by the Danish Health Authority: stroke, dehydration, constipation, pneumonia, urinary tract infection, asthma / COPD, heart failure, gastroenteritis, fracture, nutritional anaemia, arthritis, social conditions or pressure sore. We performed all analyses at 8, 30 and 180 days of follow-up after discharge.

Further, the mean number of consultations at general practitioners and visits by the general practitioner on duty during follow-up were calculated.

Municipal services were calculated as the proportion of people who received the services and the mean number of daily minutes of services during the six months after discharge. We also analysed changes in municipal services from two to four weeks before admission to the period of six months after discharge.

Finally, mortality was evaluated as an outcome.

### Baseline and follow-up measures

Data on the length of index admission, sex, and age were registered at inclusion. Baseline information on marital status at admission and country at birth were obtained through the Civil Registration System [30]. Information on department of discharge and Charlson comorbidity score [31] was obtained from the Danish National Patient Register [32]. The comorbidity index was calculated using information on primary and secondary diagnoses from all in- and outpatient contacts to hospitals in Denmark 10 years before the index admission and including the index admission.

Follow-up measures were in-hospital readmissions obtained from the Danish National Patient Register, which contains information on all contacts to Danish hospitals [32]. The register contains information on admission and discharge dates, department and hospital identification and whether the admission was elective or unplanned. Information on contacts to the general practitioner and visits by the general practitioner on duty during follow-up were obtained from the Danish National Health Service Register [33]. Information on municipal services during follow-up was obtained from the municipal registration at the three municipalities where the participants resided. The information on services from the municipality was available two to four weeks before admission and during the six months following discharge. From this registration, we calculated the referred services, divided into home practical help, home personal care and nursing, and calculated the services received per day for the whole period censoring at eventual day of death. Information on mortality was obtained from the Civil Registration System [30].

### Statistical analysis

The baseline characteristics of participants versus non-participants were compared. We performed all analyses based on the intention-to-treat principle. We analysed mortality as a descriptive measure in order to evaluate the influence of competing risk. We tested all differences using the chi-square test for binary outcomes and t-test for continuous outcomes. Logistic of binary outcomes and linear regression for continuous outcomes were used adjusting for stratifying variables (municipality and discharging hospital department). Odds ratios and β coefficients with 95% confidence intervals (95%CI) were calculated. For the primary outcome (readmissions during the first 180 days) and for mortality, we estimated the Kaplan-Meier survival plots.

The epidemiologist responsible for all statistical analysis (LCT) was blinded towards the intervention status for study participants. We performed *post-hoc* unblinded per-protocol analysis with per protocol defined as follow home visits conducted compared to control group. We used two-sided *P*-values, and ≤ 0.05 defined to be statistically significant. We performed analysis using SAS, version 9.3.

### Sample size

Using data from the Ministry of Health, among patients aged > 78 years discharged from a somatic hospital 36% was readmitted within 180 days. A sample size of 216 in each group would have a 90% power and a significance level of 5% to detect a clinical relevant reduction of 14% of readmissions from 36 to 22% in the intervention group versus the control group (the minimal relevant difference of 14% was decided based on data in a report from a Danish study, unpublished in English, but analyzed by a Danish public health institute, results can be accessed at https://www.kora.dk/media/272122/dsi-3511.pdf).

## Results

**Fig. 1:**
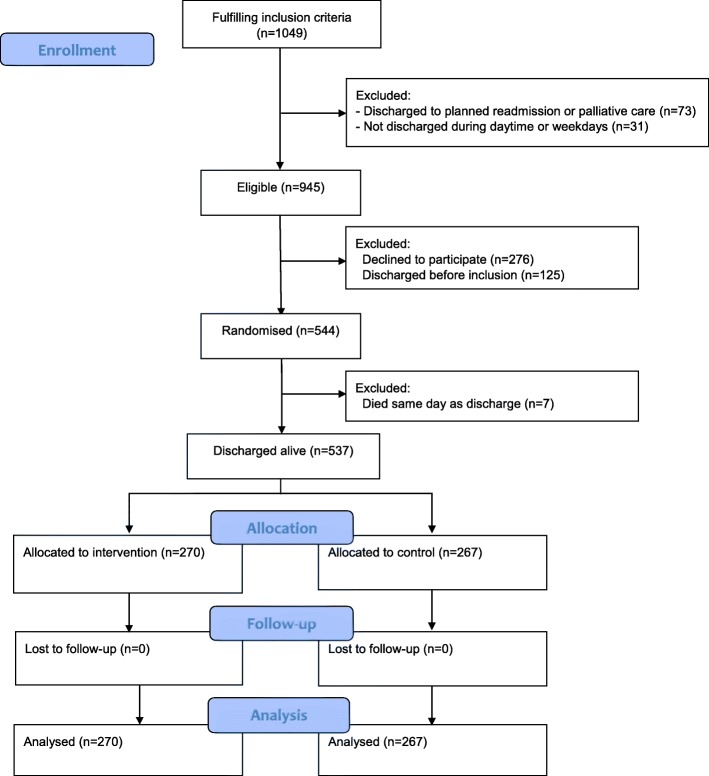
Flow chart

During the study, the age criteria were changed from 78 years to 65 years or older due to few potential participants. Of the 1049 patients fulfilling the inclusion criteria, 73 were discharged to planned readmission or palliative care, 156 were discharged before inclusion or outside daytime and 276 (23%) declined to participate (Fig. 1). Study participants were similar to eligible non-participants (data shown in Additional file 1). Seven randomized patients died on the day of planned discharge. Thus, 537 patients were randomized to intervention (*n* = 270) or control (*n* = 267). No patients were lost to follow-up because we had complete register-based outcome information on all patients (Fig. 1).

The intervention and control groups were similar in baseline characteristics with mean age of 82.5 in intervention group and 82.2 in the control group and Charlson comorbidity index of 4–11 in 26% in the intervention group and 24% in the control group, for details see Table 1.

**Table 1:** Baseline characteristics of the included patients

Baseline characteristics	Intervention (*n* = 270)	Control (*n* = 267)
Median length of stay in days before index discharge	12 (7–21)	12 (7–20)
Female gender, n (%)	152 (56)	171 (64)
Age, mean (standard deviation)	82.5 (7.6)	82.2 (7.3)
Age, n (%)		
65–69 years	23 (9)	13 (5)
70–77 years	35 (13)	51 (19)
78–84 years	97 (36)	98 (37)
85–89 years	66 (24)	59 (22)
90–102 years	49 (18)	46 (17)
Marital status, n (%)		
Married	76 (28)	77 (29)
Divorced	33 (12)	29 (11)
Unmarried	17 (6)	15 (6)
Widowed	144 (53)	146 (55)
Danish country of birth, n (%)	265 (98)	261 (98)
Charlson comorbidity score, n (%)		
0	35 (13)	43 (16)
1	55 (20)	71 (26)
2	65 (24)	51 (19)
3	44 (16)	37 (14)
4–11	71 (26)	65 (24)
Department of discharge		
Emergency Department	19 (7)	16 (6)
Geriatric Department	97 (36)	94 (35)
Surgical Department	22 (8)	23 (9)
Medical Department	97 (36)	103 (39)
Orthopedic Surgery Department	22 (8)	21 (8)
Municipality, n (%)		
Guldborgsund	129 (48)	127 (48)
Lolland	104 (39)	103 (39)
Vordingborg	37 (14)	37 (14)

544 patients were randomised, but 7 patients died on the day of discharge

In the intervention group, 238 (88%) received the planned intervention. Since discharge was planned the day before some patients became unstable and were not discharged as planned and therefore they did not get the intervention.

**Fig. 2:**
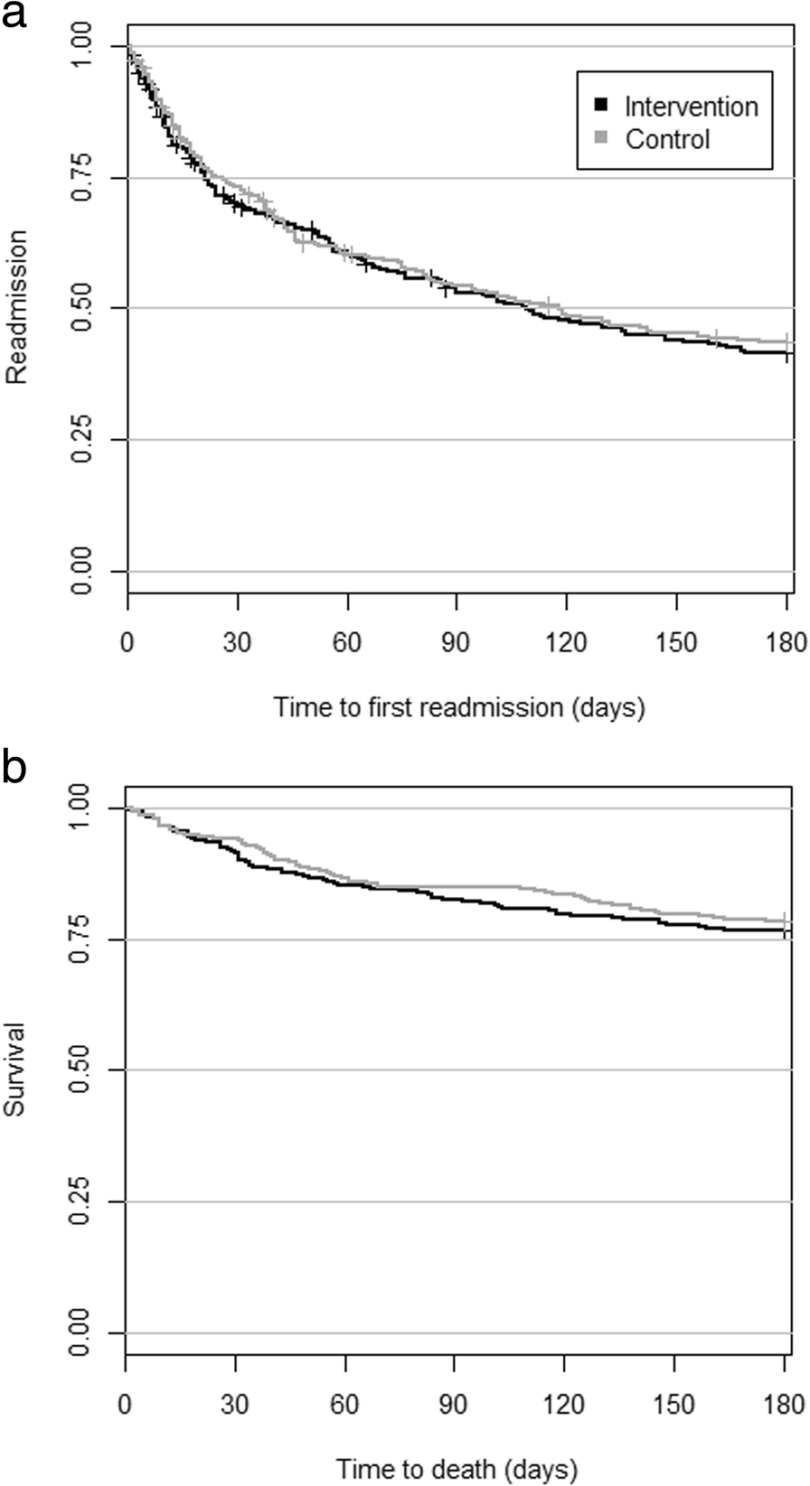
Time to **a**) first readmission and **b**) death

The intervention and control groups did not differ in the primary outcome, with 56% of intervention patients and 54% of control patients readmitted within 180 days of discharge (*p* = 0.71) (Table 2). The adjusted odds ratio was 1.07 (95%CI, 0.75–1.51). The results were similar at 8 and 30 days after discharge. The number of readmissions and length of stay were also similar at 8, 30 and 180 days. The survival curve for time until first readmission showed no difference between the two groups (Fig. 2a).

**Table 2:** Readmissions, contacts with general practitioner and deaths at 8, 30 and 180 days

	Intervention (*n* = 270)	Control(*n* = 267)	P^a^	Adjusted OR (95%CI)^b^	Adjusted β (95%CI)^c^
Number of patients readmitted, n (%)					
8 days after discharge	31 (11)	27 (10)	0.61	1.16 (0.67;2.00)	–
30 days after discharge	80 (30)	70 (26)	0.38	1.18 (0.81;1;73)	–
180 days after discharge	150 (56)	144 (54)	0.71	1.07 (0.75;1.51)	–
Total number of readmissions, sum					
8 days after discharge	32	32	0.97	–	0.00 (−0.06;0.06)
30 days after discharge	92	87	0.77	–	0.01 (−0.09;0.11)
180 days after discharge	274	293	0.49	–	−0.09 (− 0.32;0.14)
Total number of days in the hospital, sum					
8 days after discharge	81	55	0.28	–	0.09 (−0.08;0.26)
30 days after discharge	545	440	0.30	–	0.36 (−0.34;1.06)
180 days after discharge	1660	1830	0.48	–	−0.76 (−2.70;1.18)
Number of GP services, sum					
28 days after discharge	1344	1393	0.52	–	−0.26 (− 0.97;0.46)
180 days after discharge	5209	5730	0.10	–	−2.17 (−4.77;0.42)
Number of visits to GP on duty, sum					
28 days after discharge	275	267	0.56	–	0.12 (−0.25;0.49)
180 days after discharge	874	845	0.86	–	0.09 (−0.70;0.88)
Death, n (%)					
8 days after discharge	6 (2)	6 (2)	0.98	1.04 (0.33;3.30)	–
30 days after discharge	23 (9)	16 (6)	0.26	1.49 (0.77;2.89)	–
180 days after discharge	63 (23)	58 (22)	0.66	1.11 (0.73;1.66)	–

^a^Chi-square test for difference in proportions between the intervention and control groups and *t*-test for mean differences between the intervention and control groups

^b^Logistic regression of binary outcomes adjusted for discharging department and municipality, odds ratio for intervention compared to control group

^c^Linear regression of continuous outcomes adjusted for discharging department and municipality, β coefficient for intervention compared to control group

The intervention group received the same number of general practitioner services (*p* = 0.52) both at 28 days and 180 days after discharge (Table 2). The same was observed for number of visits to the general practitioner on duty. The intervention and control groups had similar mortality at 8, 30, and 180 days and the survival curve showed similar pattern (Fig. 2b). Mortality at 180 days was 23% in the intervention group and 22% in the control group.

Per-protocol analysis showed the same conclusion of no difference in readmission between groups as the intention to treat analysis.

The proportion of patients receiving municipal services was high and there were no difference between intervention and control groups (Table 3). The mean number of minutes per day and the change in minutes of care from before to after discharge was not different between intervention and control groups.

**Table 3:** Municipal services during six months after discharge

	Intervention	Control	P^a^		Adjusted OR (95%CI)^b^	Adjusted β (95%CI)^c^
Proportion receiving municipal services						
Practical help, n (%)	192 (75)	188 (72)	0.40		1.18 (0.79;1.76)	–
Personal care, n (%)	215 (84)	224 (86)	0.63		0.88 (0.54;1.43)	–
Nursing, n (%)	228 (89)	232 (89)	0.85		1.09 (0.62;1.92)	–
Minutes per day among patients receiving services						
Practical help, sum (mean)	2833 (15)	2671 (14)	0.72		–	0.41 (−2.36; 3.17)
Personal care, sum (mean)	11,364 (53)	11,079 (50)	0.55		–	3.97 (−6.63; 14.58)
Nursing, sum (mean)	4569 (20)	3746 (16)	0.21		–	4.04 (−1.93; 10.00)
Change in minutes per day						
Practical help, mean	4.3	5.2	0.62		–	−0.97 (−4.40; 2.46)
Personal care, mean	25.1	26.8	0.68		–	−1.72 (−9.11; 5.67)
Nursing, mean	11.7	8.7	0.28		–	2.97 (−2.32; 8.25)

^a^Chi-square test for difference in proportions between the intervention and control groups and *t*-test for mean differences between the intervention and control groups

^b^Logistic regression of binary outcomes adjusted for discharging department and municipality, odds ratio for intervention compared to control group

^c^Linear regression of continuous outcomes adjusted for discharging department and municipality, β coefficient for intervention compared to control group

The proportion of preventable readmissions 180 days after discharge was not different between intervention and control groups both aggregated for all preventable diagnoses and for each disease group (data shown in Additional file 2). The same result was observed for 8 and 30 days after discharge.

## Discussion

We aimed to investigate the effect of an intervention consisting of a single follow-up home visit administered to frail elderly patients at discharge after hospitalization. The intervention is not representing a new method but contributes to the evidence concerning intensity of the intervention in this patient group. We succeeded to include very vulnerable elderly patients, demonstrated by the fact that a majority was readmitted within 180 days and 23% died within 180 days. The intervention did not make any change neither concerning the primary outcome (readmission within 180 days) nor concerning any of the secondary outcomes.

### Other studies

Previous meta-analyses have found a small but significant association between different discharge planning interventions and readmission rates. A meta-analysis by Leppin et al. included 47 trials and found overall an OR of 0.82 (CI 0.73–0.91). However later studies were less likely to show effect, possibly due to improvements in overall basic care during recent years. The trials included in the meta-analysis by Leppin target different patient groups. One of the most positive studies targeted all patients who were in hospital for more than 3 days and discharged with diagnoses of gastrointestinal, cardiac or lower respiratory diseases. The intervention was not very intensive (a telephone follow up). The patients included had a mean age of 50 and were less frail than our patients [34]. Stewart et al. found effect of a single home visit. However, their patients were very different from ours with mean age around 65 years and a mean Charlson index of 1.3 [35].

Leppin et al. concluded that interventions with a greater complexity and involving several professionals seem more likely to be effective [27].

Naylor et al. showed a significant reduction in readmission rate following an intervention consisting of comprehensive discharge planning and 4 weeks follow up by trained and experienced gerontological advanced practice nurses [36].

Rytter et al. in a recent Danish trial included patients similar to ours regarding mean age, readmission rate, and mortality and found a positive effect on readmission rate. However, the intervention was far more intensive with planned and structured visits performed by the general practitioner and district nurse during the 1st, 2nd, and 8th week after discharge [37]. An older Danish study also showed a positive effect with a far more intensive follow up including several as needed visits performed by a geriatric team consisting of a specially trained nurse and a geriatrician [38].

Linertova et al. summarized findings from studies aiming to reduce admissions of elderly patients (> 75 years). Most studies did not show significant effect, but interventions including comprehensive geriatric care and home care components were most likely to have effect [39].

Compared to our study the above mentioned successful interventions were all including a more comprehensive intervention.

During several years, the health authorities in Denmark like in many other countries have focused on improving the transition to home after hospitalization of elderly patients. Several reforms stressing the need for shared responsibility and improved communication between hospital and municipality have been introduced. It is likely that these reforms have had effect and this is probably the reason that prior studies tend to be more positive than recent studies; there are no low-hanging fruits to be picked anymore.

In summary, our study includes a very frail population, situated in a low socioeconomic area and examines a minimal intervention with one home visit only. Probably a more comprehensive intervention is needed when the aim is to avoid hospitalization of frail elderly patients. This has been described as hospital-at-home intervention and analyzed in a Cochrane review from 2008. The authors conclude that a hospital-at home intervention may reduce mortality, and may increase quality of life, functional level, and cognitive abilities. The hospital at home interventions may be less expensive than in hospital care [40].

### Strengths and limitations

This study is a large randomized controlled study. Randomization was effective in securing that the control and intervention groups were similar. We have complete follow-up due to the nationwide registration. One potential limitation was that the intervention was not blinded to the project nurse, municipal nurse and patient. However, the statistician who performed all analyses was blinded to the placement of patients in control or intervention group and the interpretation of results was performed before intervention status was revealed. Using individual randomization may have caused spillover effect, i.e. staff nurses may have learned from study nurses and thereby control patients would get parts of the intervention as well. However, the study nurses who were planning discharge for intervention patients had their own working environment away from the departments and we therefore assume this possible bias to be minimal.

The assessment performed by the study nurses was structured but we did not have a strict protocol to describe the intervention that should follow a specific finding. The proper intervention relied on judgment from the nurses. This may have weakened the effect. However, the 2 nurses were experienced geriatric nurses and therefore probably capable to judge what interventions were needed in a specific patient.

The intervention was limited to patients discharged during weekdays in the daytime. However, generally only few patients from our hospital are discharged during evenings and weekends. The strength of the findings is limited by the fact that it is a single center study and therefore generalization of results should be done with caution. We do not have measures that may have been influenced by the intervention like functional level, cognitive status, and quality of life. We therefore may have missed other possible positive effects of the intervention.

## Conclusion

There is no effect of a single follow-up home visit on readmission in a group of frail elderly patients discharged from hospital. We conclude that a far more intensive intervention is needed in order to prevent readmissions among frail elderly patients.

## Supplementary information

**Additional file 1.** Baseline descriptives of included and excluded patients. The file describes the characteristics of the study participants and the eligible non-participants.

**Additional file 2.** Preventable readmissions. The file shows the proportion of preventable readmissions between the intervention and control groups, both aggregated for all preventable diagnoses and separate for each disease group (shown at 8, 30 and 180 days).

## Data Availability

It is not possible to share all the data supporting the conclusions of this article. The authors are not able to share the data, because we have used data from national registries, which we do not own. The patient data are owned by Region Zealand and are available from the authors upon reasonable request and with permission of Region Zealand.

## Abbreviations

ACSC diagnosis: Ambulatory Care Sensitive Conditions
CI: Confidence interval
COPD: Chronic obstructive pulmonary disease
OR: Odds ratio
