# Supplementary material for: Effect of single follow-up home visit on readmission in a group of frail elderly patients – a Danish randomized clinical trial
Source: BMC Health Serv Res. 2019 Oct 25;19:751. doi: 10.1186/s12913-019-4528-9 (PMC6815031; doi:10.1186/s12913-019-4528-9)
Supplement: Supplementary file 1 — Additional file 1. Baseline descriptives of included and excluded patients. The file describes the characteristics of the study participants and the eligible non-participants. [file 12913_2019_4528_MOESM1_ESM.docx]

**Additional file 1. Baseline descriptives of included and excluded patients**

| **Baseline characteristics** | Included | Not meeting inclusion criteria | Discharged to planned readmission or palliative care | Not discharged during daytime or weekdays | Declined to participate | Discharged before inclusion |
| --- | --- | --- | --- | --- | --- | --- |
| n | 544 | 170 | 73 | 31 | 276 | 125 |
| Median length of index admission (IQR) | 12 (7-21) | 10 (5-18) | 14 (8-24) | 5 (2-9) | 9 (5-16) | 7 (4-12) |
| Female gender, n (%) | 326 (60) | 87 (51) | 30 (41) | 18 (58) | 152 (55) | 67 (54) |
| Age, mean (standard deviation) | 82.4 (7.5) | 80.5 (9.3) | 80.0 (7.3) | 77.4 (9.4) | 82.4 (7.8) | 82.8 (6.8) |
| Age (n, %) |  |  |  |  |  |  |
| -64 years | 0 (0) | 5 (3) | 0 (0) | 0 (0) | 0 (0) | 1 (1) |
| 65–69 years | 37 (7) | 14 (8) | 6 (8) | 8 (26) | 24 (9) | 4 (3) |
| 70–77 years | 86 (16) | 31 (18) | 19 (26) | 8 (26) | 38 (14) | 16 (13) |
| 78–84 years | 195 (36) | 58 (34) | 27 (37) | 7 (23) | 94 (34) | 54 (43) |
| 85–89 years | 129 (24) | 38 (22) | 15 (21) | 5 (16) | 75 (27) | 30 (24) |
| 90–102 years | 97 (18) | 24 (14) | 6 (8) | 3 (10) | 45 (16) | 20 (16) |
| Marital status, n (%) |  |  |  |  |  |  |
| Married | 155 (28) | 48 (28) | 32 (44) | 8 (26) | 77 (28) | 33 (26) |
| Divorce | 63 (12) | 24 (14) | 10 (14) | 7 (23) | 30 (11) | 17 (14) |
| Unmarried | 32 (6) | 18 (11) | 1 (2) | 3 (10) | 26 (9) | 8 (6) |
| Widowed | 294 (54) | 80 (47) | 30 (41) | 13 (42) | 143 (52) | 67 (54) |
| Danish country of birth, n (%) | 533 (98) | 168 (99) | 72 (99) | 31 (100) | 274 (99) | 122 (98) |
| Charlson comorbidity score, n (%) |  |  |  |  |  |  |
| 0 | 78 (14) | 29 (17) | 3 (4) | 4 (13) | 31 (11) | 15 (12) |
| 1 | 127 (23) | 45 (26) | 7 (10) | 3 (10) | 71 (26) | 23 (18) |
| 2 | 117 (22) | 27 (16) | 10 (14) | 10 (32) | 61 (22) | 37 (30) |
| 3 | 83 (15) | 19 (11) | 17 (23) | 3 (10) | 44 (16) | 25 (20) |
| 4–15 | 139 (26) | 50 (29) | 36 (49) | 11 (35) | 69 (25) | 25 (20) |
