# Supplementary material for: Effect of single follow-up home visit on readmission in a group of frail elderly patients – a Danish randomized clinical trial
Source: BMC Health Serv Res. 2019 Oct 25;19:751. doi: 10.1186/s12913-019-4528-9 (PMC6815031; doi:10.1186/s12913-019-4528-9)
Supplement: Supplementary file 2 — Additional file 2. Preventable readmissions. The file shows the proportion of preventable readmissions between the intervention and control groups, both aggregated for all preventable diagnoses and separate for each disease group (shown at 8, 30 and 180 days). [file 12913_2019_4528_MOESM2_ESM.docx]

**Additional file 2**. **Preventable readmissions**

|  | 8 days | | | 30 days | | | 180 days | | |
| --- | --- | --- | --- | --- | --- | --- | --- | --- | --- |
|  | Intervention | Control | p | Intervention | Control | p | Intervention | Control | *p* |
| **Preventable diagnosis (all)** | 8 (3.0) | 4 (1.5) | 0.25 | 23 (8.5) | 18 (6.7) | 0.43 | 59 (21.9) | 59 (22.1) | 0.95 |
|  |  |  |  |  |  |  |  |  |  |
| **Stroke** | 1 (0.4) | 0 (0.0) | 0.32 | 3 (1.1) | 0 (0.0) | 0.08 | 5 (1.9) | 4 (1.5) | 0.75 |
| **Dehydration** | 0 (0.0) | 0 (0.0) | - | 1 (0.4) | 2 (0.8) | 0.56 | 4 (1.5) | 5 (1.9) | 0.72 |
| **Constipation** | 0 (0.0) | 0 (0.0) | - | 0 (0.0) | 0 (0.0) | - | 1 (0.4) | 3 (1.1) | 0.31 |
| **Pneumonia** | 2 (0.7) | 1 (0.4) | 0.57 | 9 (3.3) | 3 (1.1) | 0.08 | 22 (8.2) | 15 (5.6) | 0.25 |
| **Urinary tract infection** | 1 (0.4) | 0 (0.0) | 0.32 | 2 (0.7) | 3 (1.1) | 0.64 | 6 (2.2) | 8 (3.0) | 0.57 |
| **Chronic obstructive lung disease** | 0 (0.0) | 0 (0.0) | - | 3 (1.1) | 2 (0.8) | 0.66 | 8 (3.0) | 10 (3.8) | 0.61 |
| **Ischemic heart disease** | 3 (1.1) | 2 (0.8) | 0.75 | 3 (1.1) | 3 (1.1) | 0.99 | 7 (3.6) | 11 (4.1) | 0.33 |
| **Gastroenteritis** | 0 (0.0) | 0 (0.0) | - | 0 (0.0) | 0 (0.0) | - | 0 (0.0) | 0 (0.0) | - |
| **Fragility fracture** | 0 (0.0) | 0 (0.0) | - | 2 (0.7) | 2 (0.8) | 0.99 | 8 (3.0) | 5 (1.9) | 0.41 |
| **Iron deficiency anemia** | 0 (0.0) | 0 (0.0) | - | 0 (0.0) | 0 (0.0) | - | 1 (0.4) | 1 (0.4) | 0.99 |
| **Arthritis** | 0 (0.0) | 0 (0.0) | - | 0 (0.0) | 0 (0.0) | - | 1 (0.4) | 1 (0.4) | 0.99 |
| **Social causes** | 1 (0.4) | 1 (0.4) | 0.99 | 1 (0.4) | 2 (0.8) | 0.56 | 3 (1.1) | 2 (0.8) | 0.66 |
| **Pressure ulcers** | 0 (0.0) | 0 (0.0) | - | 0 (0.0) | 1 (0.4) | 0.31 | 1 (0.4) | 1 (0.4) | 0.99 |
